# Socially Aversive Personality and the symptoms of Body Dysmorphic Disorder in the Korean Young Adult population

**DOI:** 10.1192/j.eurpsy.2023.794

**Published:** 2023-07-19

**Authors:** S. Han, H.-S. Chee

**Affiliations:** 1 Psychiatry, Chungnam National University Hospital; 2 Institute of Brain Research, Chungnam National University, Daejeon, Korea, Republic Of

## Abstract

**Introduction:**

Body dysmorphic disorder (BDD) is defined in DSM5 as a preoccupation with one or more perceived defects or flaws in physical appearance causing significant distress or impairment in social and occupational functioning. Despite many studies on mental health disorders related to BDD, the diagnosis is still frequently overlooked.

**Objectives:**

Previous studies have examined the general personality characteristics of BDD. The objective of this study is to find out how socially aversive personality traits are related.

**Methods:**

Total of 86 mentally and physically healthy adults participated. BDD was assessed by BDDE-SR, and aversive personality was assessed by Short Dark Triad (SD3: Machiavellianism, narcissism, psychopathy), Assessment of Sadistic Personality (ASP), and paranoid (PAR), borderline (BOR), and antisocial (ANT) features of the clinical subscales of Personality Assessment Inventory(PAI). Correlations between the reported scores were investigated using Pearson’s and regression was performed on relevant scales.

**Results:**

Thirty seven males and 49 females (mean age 23.8 years) showed no statistically significant difference in total BDDE-SR was reported based on sex(p=0.18) or BMI(underweight, normal, overweight, p=0.236). BDDE-SR, SD3 and ASP were not statistically correlated, but all of the subscales of PAR(PAR-H, PAR-P, PAR-R), BOR(BOR-A, BOR-I, BOR-N, BOR-S) and ANT(ANT-A, ANT-E, ANT-S) were associated with BDDE-SR. Regression results demonstrated in Table 1 show that BOR-I and PAR-R predict BDDE-SR. Correlation of BOR-I and PAR-R with BDDE-SR factors was shown in Table 2.

**Table 1.** Hierarchical multiple linear regression analysis for BOR-I, PAR-R in predicting BDD symptoms

	B	SE	*beta*	t	R²	ΔR²	F	Sig.
Model 1								
BOR-I	2.812	0.547	0.491	5.140	.241	.232	26.421	.001
Model 2								
BOR-I	2.317	0.568	0.405	4.080	.294	.277	6.073	.016
PAR-R	1.387	0.563	0.245	2.464

**Table 2.** Correlation between BDDE Total, five BDDE factors and BOR-I and PAR-R

Variable	BDDE Total	Factor 1	Factor 2	Factor 3	Factor 4	Factor 5	BOR-I
BOR-I	.489**	.469**	.370**	.440**	.317**	.352**	
PAR-R	.388**	.311**	.366**	.307**	.302**	.387**	.354**

*Note.*

** p<0.01;

Factor 1 : preoccupation, distress and embarrassment; Factor 2 : avoidance; Factor 3 : checking, comparing and camouflaging; Factor 4 : dissatisfaction; Factor 5 : importance; BOR-I : identity problem of borderline traits; PAR-R : resentment of paranoid traits

**Conclusions:**

This study shows that BDD symptoms are associated with borderline-identity problems and paranoia-resentment and suggests that we should consider the diagnosis of BDD for individuals with high BOR and PAR scores.

**Disclosure of Interest:**

None Declared

